# Social disparities, health risk behaviors, and cancer

**DOI:** 10.1186/1471-2482-13-S2-S17

**Published:** 2013-10-08

**Authors:** Stefania Rametta, Giuseppe Grosso, Fabio Galvano, Antonio Mistretta, Stefano Marventano, Francesca Nolfo, Silvio Buscemi, Santi Gangi, Francesco Basile, Antonio Biondi

**Affiliations:** 1 Department “G. F. Ingrassia” Section of Hygiene and Public Health, University of Catania, Catania, Italy; 2 Department of Drug Sciences, Section of Biochemistry, University of Catania, Catania, Italy; 3 Department of Internal Medicine, University of Palermo, Palermo, Italy; 4 Department of General Surgery, Section of General Surgery and Oncology, University Medical School of Catania, Italy

## Abstract

**Background:**

Overall cancer incidence rates decreased in the most recent time period in both men and women, largely due to improvements in surgical therapeutic approaches (tertiary prevention) and screening programs (secondary prevention), but differences in cancer incidence and survival according to socioeconomic status are documented worldwide. Health risk behaviors, defined as habits or practices that increase an individual’s likelihood of harmful health outcomes, are thought to mediate such inequalities.

**Discussion:**

Obesity has been related with increased cancer incidence and mortality due to imbalance of leptin and adiponectin which are connected to activation of PI3K, MAPK, and STAT3 pathways and decreasing insulin/insulin-like growth factor (IGF)-1 and mTOR signaling via activation of 5 AMP-activated protein kinase (AMPK), respectively. Physical activity has been associated to prevent cancer by the aforementioned obesity-related mechanisms, but also increasing level of circulating vitamin D, which has been related to lower risk of several cancers, and increasing prostaglandin F2a and reducing prostaglandin E2, which are both related with cancer prevention and promotion, respectively. A large number of different substances may induce themselves a direct cytotoxicity and mutagenic action on cells by smoking, whereas alcohol promote immune suppression, the delay of DNA repair, inhibition of the detoxification of carcinogens, the production of acetaldehyde, and the contribution to abnormal DNA methylation. The combined smoking and alcohol drinking habits have been shown to increase cancer risk by smoke action of increasing the acetaldehyde burden following alcohol consumption and alcohol action of enhancing the activation of various procarcinogens contained in tobacco smoke.

**Conclusions:**

Interventions at the social level may be done to increase awareness about cancer risks and promote changing in unhealthy behaviors.

## Background

Cancer is a leading cause of death worldwide, accounting for 7.6 million deaths (around 13% of all deaths) [1]. Overall cancer incidence rates decreased in the most recent time period in both men and women, largely due to decreases in the 3 major cancer sites in men (lung, prostate, and colorectum) and 2 major cancer sites in women (breast and colorectum) [2]. It has been documented that a decrease has been reached also in mortality rates, mostly due to improvements in surgical therapeutic approaches [3-5]. However, different outcomes have been reported due to non-modifiable factors such age [6,7] and increased cancer mortality due to modifiable factors, such as the socioeconomic status, has been well documented worldwide, irrespectively of national health-care system [8-10]. Social disparities in cancer survival are multidimensional and may depend on factors related to the public health care organization [11]. These factors may regard screening, diagnosis conditions, access to specialized care, treatment or follow-up modalities, and they vary according to the health care systems [12]. In countries where the insurance status is crucial for access and continuity of care, increased financial resources may support patients to better manage the disease [13,14]. Many evidences have demonstrated inequalities by socioeconomic status and race. Several studies have reported inequalities at different levels, for instance in delay of hospitalization or advanced cancer stage at diagnosis for disadvantaged groups [15,16]. Similar trends have been reported regarding survival of cancer patients, evidencing inequalities among lower socioeconomic classes and economically disadvantaged race and ethnicity groups [17,18]. Cultural disparities may depend on a different access to health information. In example, higher education has been associated with increased internet use and high eHealth literacy which is related to have increased knowledge and previous screening practice related to colorectal cancer compared to those with low eHealth literacy [19]. A higher education and knowledge about colorectal cancer related information has been also related with an increased acceptance of colorectal cancer screening programs [20]. On the other hand, in countries with equal access to health-care facilities, a direct economic hindrance in seeking medical health care cannot be relevant, because health-care facilities are tax-financed. Thus, socioeconomic and cultural status may act by psychosocial pathways. More acculturated people may have higher knowledge about health-related topics, be more aware of their symptoms and communicate better with health staff than low-cultural people [21,22]. Health risk behaviors are defined as habits or practices that increase an individual’s likelihood of harmful health outcomes. They are thought to explain, at least in part, many social inequalities in health status of populations. It has been reported that health risk behaviors, including diet, physical activity, and smoking, explain the higher frequencies of several cancers and mortality among those of lower socio-economic status [23]. On the other hand, although many of these factors are modifiable causes of cancer, it is challenging to plan interventions acting on the specific associations between them and cancer over a lifetime, due to the long latent period for cancer development and its complex pathogenesis. Thus, the only possibility to prevent cancer is to increase awareness in people regarding health-related behaviors in order to establish life-long habits that may decrease the risk of developing malignancies. As most of cancer-promoting factors are related with the social status of one person, we will discuss of the main well-recognized cause of cancer which may mediate social and cultural effects on cancer developing and survival.

## Obesity

Obesity has dramatically increased during the last few decades both in developed and now also in developing countries, contributing to the global rising of cardiovascular diseases [24]. Nowadays, it has been estimated that overweight population increased in European countries ranging between 8% and 40% in men and between 5% and 53% in women [25], whereas in the United States reached the 66% of adults with a BMI >25 kg/m2 and half of those have a BMI of >30 kg/m2 [26]. The fast rise of obesity in Western countries cannot be related with a genetic mutation due to the high rapidity of the phenomenon. Conversely, over the past few decades, the transformation of the modern environment leaded to changes in diet and physical activity.

Obesity has been widely associated with non-communicable diseases, such as cardiovascular diseases (heart disease and stroke), diabetes, osteoarthritis and musculoskeletal disorders, fatty liver, gall stones, psychological disorders, and psychosocial problems [27] as well as it has been also related to increased mortality [28]. Obesity has been also associated to a higher incidence of many cancers, including cancers of the endometrium, kidney, gallbladder (in women), breast, colon, and esophagus [29], and increased cancer-related mortality [30]. An indirect association between obesity and diet quality may explain the increased risk of cancer [30,31], since the lacking of healthy nutrients may occurs in subjects consuming low-quality diet [31-36]. On the other hand, despite the biological mechanisms explaining the direct relationship between obesity and cancer are still unclear, several hypotheses have been proposed [37]. As adipose tissue is an endocrine organ that produces and secretes polypeptide hormones (i.e., leptin and adiponectin), it has been hypothesized that imbalance of production of such hormones may be involved in cancer development [38,39]. Specifically, a pro-carcinogenic effect of leptin has been demonstrated by activation of PI3K, MAPK, and STAT3 pathways [40-42] whereas adiponectin may exert anticancer effects by decreasing insulin/insulin-like growth factor (IGF)-1 and mTOR signaling via activation of 5 AMP-activated protein kinase (AMPK) and exerting anti-inflammatory actions via the inhibition of nuclear factor kappa-light-chain-enhancer of activated B cells (NF-κB) [43]. Also steroid hormones, including estrogen, progesterone, androgens, and adrenal steroids are associated with adipose tissue [44] and may play a role on progression of several types of male and female cancer [45].

An inflammation theory for cancer development related to obesity has been also hypothesized. Indeed, the increased levels of proinflammatory cytokines and various interleukins related to body adiposity, may stimulate the activation of NF-κB complex which may promote cancer development itself [46].

Hyperinsulinemia and elevated IGF-1 are related to the diabetic condition and the obese status. Both insulin and IGF-1 have been hypothesized to play a role on cancer promotion through the Akt/PI3K/mTOR cascade that promotes cell growth and proliferation [47,48]. On the contrary, caloric restriction induce the disruption of the Akt/PI3K/mTOR cascade at least in part via AMPK activation [49,50] and is frequently associated with a decreased cancer incidence of breast cancer in humans and in animal models [51,52]. Interestingly, similar pathways are involved in cancer promotion and progression irrespectively of the primary cause, thus suggesting a possible target for therapy [53,54].

Obesity has been related also to decreased survival in patients affected by several types of cancers [55,56] although no study has elucidated the causal mechanism and there is currently no evidence that weight loss after diagnosis improves survival. Considerations regarding obese patients are focused on chemotherapy, radiotherapy, and surgical treatment [34]. Concerns of relative over- (due to increased weight) or under-dosing (to avoid toxicity) of chemotherapy in the obese cancer patients have been reported [57]. Moreover, technical difficulties in positioning obese patients during radiotherapy may occur [58]. Finally, high BMI has been strongly, but not univocally, predictive of worse operative outcomes [59].

## Physical activity

A protective association between physical activity and colon, breast, ovarian, lung, and renal cancers is supported by a number of review articles [60-67]. Similar effects have been demonstrated also in prolonging survival in cancer patients [68]. Unlike these consistently observed findings, the association with rectal cancer is still uncertain [69] maybe due to the different carcinogenic mechanisms related with the cancer location. Main hypothesized mechanisms include those aforementioned obesity-related such as decreased adipose tissue accumulation, decreased inflammation, reduced levels of insulin and IGF- 1 and modulated immune response [70]. Physical activity also increase level of circulating vitamin D [71], which has a direct anti-carcinogenic effect on colonic epithelial cells [72] and has been related to lower risk of colon, renal, and other cancers [73-75]. Moreover, the decreased bowel transit time induced by physical activity reduce the exposure of the colon to colonic contents, bile acids and other potential carcinogens [76]. Finally, physical activity has been also related to increased prostaglandin F2a [77] and reduced prostaglandin E2 [78] that are both related with cancer prevention and promotion, respectively [79].

## Smoking and alcohol drinking

Several reports seem to demonstrate the detrimental effects of smoke on health, increasing risk of many cancers, including lung, laryngeal and pharyngeal, followed by upper digestive tract and oral cancers [80], as well as bladder [81] renal [82], breast [83], and colorectal cancers [84]. Despite the pathogenicity of tobacco smoking for pulmonary and urologic cancers appears well understood, doubts on the precise biological mechanisms on colorectal cancer promotion and progression still exist. The way by which cigarette smoking may induce lung malignancy includes a large number of different substances, most of them currently unknown, that may induce themselves a direct cytotoxicity and mutagenic action on lung epithelial cells by means of generation of DNA mutations, epigenetic events, epithelial cell to mesenchymal cell transformations, as well as by chronic cell damage [85,86]. Regarding low digestive tract cancers, epidemiological data revealed that a long period of exposure is required to increase risk of colon cancer [87]. It has been hypothesized that the possibility of proto-oncogene mutation in gastrointestinal mucosa cells may be associated with tobacco smoking-induced cancers through the formation of unfavorable DNA adducts [88]. Moreover, the association of smoking with rectal cancer seems to be stronger than with colon [87,89].

Alcohol has been reported to cause nearly 4% of the global cancer burden [90], and chronic consumption has been associated with cancers of the oral cavity, larynx, pharynx, esophagus, liver, colon, rectum, and breast [91]. Some meta-analyses of case-control [92] and cohort studies [93-95] concluded that a daily alcohol intake of 25-30 g or more is significantly associated with increased risk of colon and rectal cancer, suggesting a linear dose-response relationship. The mechanisms hypothesized to play a role in cancer promotion involve the immune suppression, the delay of DNA repair, the induction of cytochrome P-450 enzymes that inhibit the detoxification of carcinogens (including nitrosamine), the changes in bile acid composition, the production of acetaldehyde (a known carcinogen implicated in colorectal carcinogenesis), and the contribution to abnormal DNA methylation [96,97]. Moreover, alcohol may enhance the penetration of other carcinogenic molecules into mucosal cells by acting as a solvent and may stimulate regenerative cell growth by various cytotoxic mechanisms including the excess production of oxygen free radicals [97].

The combined smoking and alcohol drinking habits have been shown to be detrimental for health and notably increase cancer risk by smoke action of increasing the acetaldehyde burden following alcohol consumption and alcohol action of enhancing the activation of various procarcinogens contained in tobacco smoke due to increased metabolic activation by an induced cytochrome P450-2E1-dependent microsomal biotransformation system in the mucosa of the upper digestive tract and the liver [98].

## Recommendations and conclusions

The most recognized interventions on cancer prevention regard secondary prevention, such as screening programs. These interventions aim to diagnose the malignancies at an early stage and to treat these lesions before spread occurs. On the other hand, they do not interfere with factors which may play a role in the genesis and promotion of the disease. Primary prevention may reduce the exposure to cancer-promoting environmental and behavioral influences [99].

A series of intervention through public health policy can be made in order to decrease cancer risk among general population. Regarding smoking habit, a six-point intervention list has been developed by the World Health Organization and focus on the following main features: monitor tobacco use and prevention policies; protect people from tobacco smoke; offer help to quit tobacco use; warn about the dangers of tobacco; enforce bans on tobacco advertising, promotion, and sponsorship; and raise taxes on tobacco [100]. Public policy options for alcohol control include, as well as with tobacco control, availability and taxation, for example by reducing retail hours and density of alcohol outlets, which has been reported to reduce sales and consumption [101-103]. Moreover, interventions at the social level may be done to discourage consumption. Public health efforts should be made in order to ameliorate the environmental context for healthy eating by providing easier access and price incentives for healthy foods such as fruit and vegetables. Interventions on food advertising have been demonstrated to be effective in increase diet quality and reduce obesity rates [104]. Moreover, education on maximizing opportunities for physical activity, such as encouraging stair use over elevators, may help people to maintain a healthy weight.

## Competing interests

The authors declare that they have no competing interests.

## Authors' contributions

SR: conception and design, drafting the manuscript; GG, AM, SM, FN: drafting the manuscript; SB, FG, FB, SG, AB: critical revision, given final approval of the version to be published.

## Declarations

Funding for this article came from University funds.

This article has been published as part of *BMC Surgery *Volume 13 Supplement 2, 2013: Proceedings from the 26th National Congress of the Italian Society of Geriatric Surgery. The full contents of the supplement are available online at http://www.biomedcentral.com/bmcsurg/supplements/13/S2
